# Dark Field and Coherent Anti-Stokes Raman (DF-CARS) Imaging of Cell Uptake of Core-Shell, Magnetic-Plasmonic Nanoparticles

**DOI:** 10.3390/nano11030685

**Published:** 2021-03-09

**Authors:** Grace Brennan, Sally Ryan, Tewfik Soulimane, Syed A. M. Tofail, Christophe Silien

**Affiliations:** 1 Department of Physics and Bernal Institute, University of Limerick, V94 T9PX Limerick, Ireland; Grace.Brennan@ul.ie (G.B.); Tofail.Syed@ul.ie (S.A.M.T.); 2 Department of Chemical Sciences and Bernal Institute, University of Limerick, V94 T9PX Limerick, Ireland; Sally.Ryan@ul.ie (S.R.); Tewfik.Soulimane@ul.ie (T.S.)

**Keywords:** magnetic-plasmonic nanoparticles, coherent anti-Stokes Raman (CARS), cell imaging, nonlinear optics, contrast agents, z-scan, biocompatibility, nanoparticles, multimodal imaging

## Abstract

Magnetic-plasmonic, Fe_3_O_4_-Au, core-shell nanoparticles are popular in many applications, most notably in therapeutics and diagnostics, and thus, the imaging of these nanostructures in biological samples is of high importance. These nanostructures are typically imaged in biological material by dark field scatter imaging, which requires an even distribution of nanostructures in the sample and, therefore, high nanoparticle doses, potentially leading to toxicology issues. Herein, we explore the nonlinear optical properties of magnetic nanoparticles coated with various thicknesses of gold using the open aperture z-scan technique to determine the nonlinear optical properties and moreover, predict the efficacy of the nanostructures in nonlinear imaging. We find that the magnetic nanoparticles coated with gold nanoseeds and thinner gold shells (ca. 4 nm) show the largest nonlinear absorption coefficient β and imaginary part of the third-order susceptibility Im χ^(3)^, suggesting that these nanostructures would be suitable contrast agents. Next, we combine laser dark field microscopy and epi-detected coherent anti-Stokes Raman (CARS) microscopy to image the uptake of magnetic-plasmonic nanoparticles in human pancreatic cancer cells. We show the epi-detected CARS technique is suitable for imaging of the magnetic-plasmonic nanoparticles without requiring a dense distribution of nanoparticles. This technique achieves superior nanoparticle contrasting over both epi-detected backscatter imaging and transmission dark field imaging, while also attaining label-free chemical contrasting of the cell. Lastly, we show the high biocompatibility of the Fe_3_O_4_ nanoparticles with ca. 4-nm thick Au shell at concentrations of 10–100 µg/mL.

## 1. Introduction

Magnetic-plasmonic nanoparticles have been widely studied for nanotheranostic applications, including hyperthermia (photothermal and magnetic), drug delivery, surface-enhanced Raman scattering, and magnetic resonance image contrasting. Such nanostructures are interesting as optical contrast agents as a replacement for fluorophores, owing to their photostability, biocompatibility, and enhanced optical properties owing to the localized surface plasmon. In this work, we will examine a combined dark field and nonlinear coherent anti-Stokes Raman (CARS) optical imaging technique for visualization of the intercellular uptake of magnetic-plasmonic nanoparticles.

Dark field optical microscopy is a common method that excludes unscattered light from the image so that light scattering objects appear bright on a dark background. Typically, this technique employs a broadband light source focused onto the sample using a high numerical aperture (NA) condenser with a central block, producing a hollow cone of light. In transmission, a lower NA objective collects only scattered light. Broadband, white light dark field microscopy (and spectroscopy) is a very popular and powerful tool for the study of plasmonic nanoparticles alone [1] and in cells [2]. With a suitable choice of objective, the central beam stop may instead be placed in the collection path, collecting high scattering angles instead. This configuration is useful in laser-based dark field microscopy as the laser does not need excessive expansion, but considerable light scatter is lost in the low angles to the beam stop. Laser-based dark field microscopy has also been demonstrated in several configurations, including grazing [3]/diagonal [4] incidence, total internal reflection [5]/vertical illumination [6], using an axicon lens pair [7], and using beam blocks at the objective or in the Fourier plane [8,9]. Applications include microsecond nanometer detection [6], flow cytometry [10], monitoring cellular organelle transport [11], and using supercontinuum laser for high-speed spectroscopy [12]. Plasmonic nanostructures are commonly visualized in cells and tissues by broadband dark field microscopy, however, this technique relies on a homogenous distribution of nanoparticles within cell or tissue, as the contrast in the image originates from the nanoparticles alone as opposed to the biological sample.

Coherent anti-stokes Raman scattering (CARS) microscopy is a label-free, nonlinear chemical imaging technique that can be used to probe specific intrinsic molecular vibrations of a sample. Two laser beams are used (Stokes and pump) that are tuned with a frequency difference to match with the vibrational resonance of the molecule of interest. In biological imaging, the excitation lasers can be tuned to resonate with the vibration of lipids, proteins, and even DNA without requiring fluorescent labeling. Furthermore, CARS signals can be enhanced by plasmons [13,14]. A downfall of CARS is a reasonably high background signal, this can be improved by adding modulation and demodulation in stimulated Raman scattering or by using different detection techniques like epi (backward) detection [15] or even annular [16], i.e., “dark field” collection. Gold nanoparticles have been used to locally enhance CARS signal [17], and silica nanoparticles with gold nanoshells have shown contrast in two-photon-induced photoluminescence [18] and epi-detected four-wave mixing [19]. Moreover, the third-order nonlinear optical response of Fe_3_O_4_ nanocubes has been shown to be enhanced by the addition of a silver nanoparticle, owing to the plasmon resonance [20]. This enhanced nonlinear optical response of Fe_3_O_4_-Ag, nanocube-nanosphere dimer nanoparticles indicates that magnetic-plasmonic nanoparticles in this work may be effective CARS contrast agents.

There are drawbacks associated with the current bioimaging techniques of magnetic plasmonic nanostructures. First, optical scatter imaging of such nanostructures is optimal in the visible light range as the scattering and absorption resides in this region [21]. Moreover, imaging at this wavelength risks nanoparticle heating due to proximity to absorption peak, and in biological samples, light penetration depth is not optimal. Secondly, a gold shell likely leads to reduced MRI contrasting capability. Furthermore, in optical imaging, contrast arises from nanoparticles not the biological material, hence large concentrations of nanoparticles are needed for uniform nanoparticle distribution. While in MRI imaging, contrasting is important to distinguish diseased tissue.

Herein we will explore the use of nonlinear optical microscopy techniques to image the uptake of magnetic-plasmonic nanoparticles in pancreatic cancer cells. Human pancreatic cancer cells are exposed to magnetic-plasmonic nanoparticles consisting of a ca. 20-nm diameter Fe_3_O_4_ nanoparticle coated with a thin ca. 4-nm gold shell, as reported elsewhere [21]. These nanoparticles are internalized in the cells, fixed, and subsequently imaged by various single beam imaging techniques (bright field, dark field and epi-detected backscatter) and by epi-CARS. Epi-CARS imaging is performed in the near-infrared, and the image contrast arises from both the cells and nanoparticles. We explore the nonlinear absorption coefficient β and imaginary part of the third-order susceptibility Im χ^(3)^ of magnetic-plasmonic nanostructures with various thicknesses of gold shell using open aperture z-scan techniques. Lastly, we report on the biocompatibility of the magnetic-plasmonic nanoparticles using MTT assays. 

## 2. Materials and Methods

### 2.1. Laser Microscopy Setup

**Figure 1:**
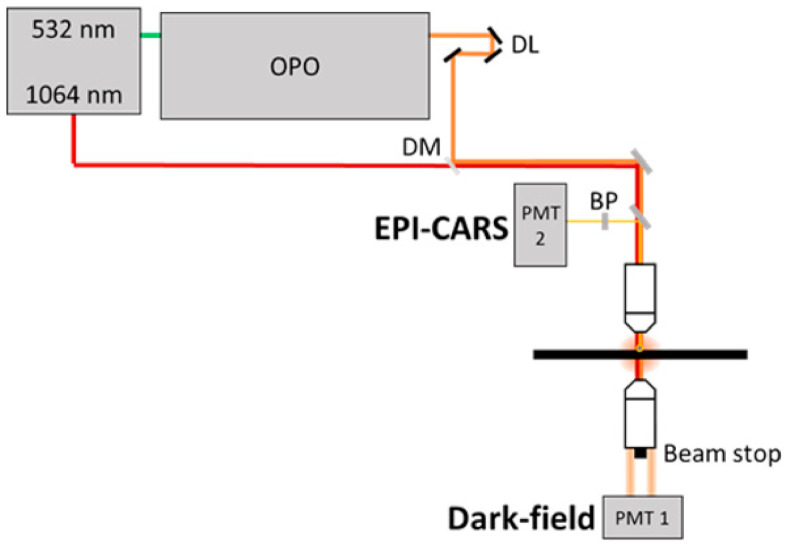
Combined dark field (DF) and coherent ant-stokes Raman (CARS) microscopy setup composed of a laser optical parametric oscillator (OPO) system, delay line (DL), dichroic mirror (DM), objectives, beam stop, bandpass filter (BP), and photomultiplier tube detection (PMT).

A picosecond fiber laser (Antares, Spark Lasers, Martillac, France) produces a 1064 nm signal (80 MHz, 6 ps), used as the Stokes beam. The second harmonic of the fiber laser at 532 nm pumps an optical parametric oscillator (Levante Emerald, APE, Berlin, Germany). The optical parametric oscillator has a tunable output that is used as the pump beam (690–990 nm), thus Raman shifts from 700 to 5000 cm^−1^ may be probed. These beams are temporally and spatially overlapped at the sample using a series of telescopes and mirrors and are combined using a dichroic mirror (DMLP905, Thorlabs, NJ, USA). A homebuilt transmission microscope is used (illumination BF MPlanFL 0.8 numerical aperture (NA), Olympus, Tokyo, Japan with iris diaphragms to select NA, Olympus DF UMPlanFl 0.8 NA in transmission), with CARS signal collected in reflection (epi-detection) using a photomultiplier tube (H11901-20, Hamamatsu, Shizuoka, Japan) using a short pass dichroic mirror (638 nm cutoff, DMSP638R, Thorlabs) and bandpass filter (650/40, FB650-40, Thorlabs). In transmission, a homemade circular beam stop (Ø 14 mm) was used to block non-scattered light and collect the dark field signal. The sample was scanned up to 100 × 100 µm using a piezoelectric scanner stage (NanoCube^®^ XYZ piezo stage, Physik Instrumente, Karlsruhe, Germany ) interfaced using a DAQ (BNC-2110, National Instruments, TX, USA) and a custom LabView program. See Figure 1 for the experimental scheme. Furthermore, this multimodal system could be modified to carry out additional microscopy techniques such as second harmonic generation, third harmonic generation, and four-wave mixing.

### 2.2. Z-Scan Technique

A simple open aperture z-scan setup was used to study the nonlinear optical properties of magnetic-plasmonic nanoparticles with different thicknesses of gold on the magnetic, Fe_3_O_4_ nanoparticle core. The methodology employed by Salah et al. [22] to study CdSe-Au nanocrystals is applied in this work. The aforementioned optical parametric oscillator laser system is used, with the output tuned to 690 nm to probe as close as possible to the absorption of the nanostructure. A 50-mm lens focuses the laser onto the sample, which is manually translated, and the transmitted signal is refocused using a lens onto a Si Detector (Thorlabs) integrated into a custom LabVIEW program (2009, Version 9.0.1) with 200 data points per measurement. The laser is blocked between measurements to minimize thermal effects.

### 2.3. Cell Culturing, Nanoparticle Viability Assays and Preparation for Imaging

Human pancreatic cancer cells (KLM-1, RIKEN Cell Bank, Ibaraki, Japan) were cultured in RPMI-1640 media supplemented with 2 mM L-glutamine, 1.5 g/L sodium bicarbonate, 4.5 g/L glucose, 10 mM HEPES, 1.0 mM sodium pyruvate, and 10% fetal bovine serum; and maintained at 37 °C in a 5% CO_2_ incubator. A gemcitabine resistant cell line GR-KLM-1 was established by exposing KLM1 cells to gemcitabine, as described previously [23].

In exposure dose studies, cells were plated in 96-well plates at seeding densities of 0.01 × 10^6^ cells/well. After incubation overnight, media was removed and replaced with media containing nanoparticles at concentrations of 10–100 ug/mL for 48 h. After the treatment period, media containing nanoparticles was removed and the cells were rinsed PBS before the addition of 100 µL of serum-free media and 10 µL of MTT solution (5 g/L in PBS) to each well for a 2 h incubation at 37 °C. One-hundred microliters of solubilization buffer (10% SDS in 0.01 M HCl) was added to each of the wells and further incubated for 4 h at 37 °C. The optical density at 570 nm was determined spectrophotometrically with a reference wavelength of 630 nm. As absorbance is proportional to cell viability, the percentage of viable cells was calculated after treatment as a percentage of cell viability compared to untreated controls. For each nanoparticle concentration, 36 plates were analyzed.

For cell imaging after exposure to nanoparticles, cells were seeded at densities of 1 × 10^6^ cells on sterile glass slides in 100-mm petri dishes. After overnight incubation, cells were subsequently exposed to the magnetic-plasmonic nanoparticles at a concentration of ca. 100 mg/mL within the cell culture media for 48 h. Cells were rinsed with PBS and fixed with 4% paraformaldehyde in PBS for 10 min at room temperature before storing in PBS. Glass slides were removed from solution and subsequently imaged as described in Section 2.1.

## 3. Results

The magnetic-plasmonic nanoparticles used in this work have been reported previously; a comprehensive synthesis protocol and transmission electron microscopy can be found in [21], the nanoparticles were produced by one gold reduction on gold-seeded iron oxide nanoparticles and are referred to as the R1 stage consisting of a ca. 20 nm diameter Fe_3_O_4_ nanoparticle coated with a thin ca. 4-nm gold shell. The magnetic-plasmonic nanoparticles can provide image contrast in laser dark field microscopy [21] and MRI imaging [24]. These nanostructures may generate stimuli-induced heating through laser illumination [21] or through exposure to an alternating magnetic field [24]. Moreover, the magnetic nature of the nanoparticle enables active targeting of targeted tissue using magnetic fields, and the gold shell also facilitates further functionalization with targeting molecules [25].

**Figure 2:**
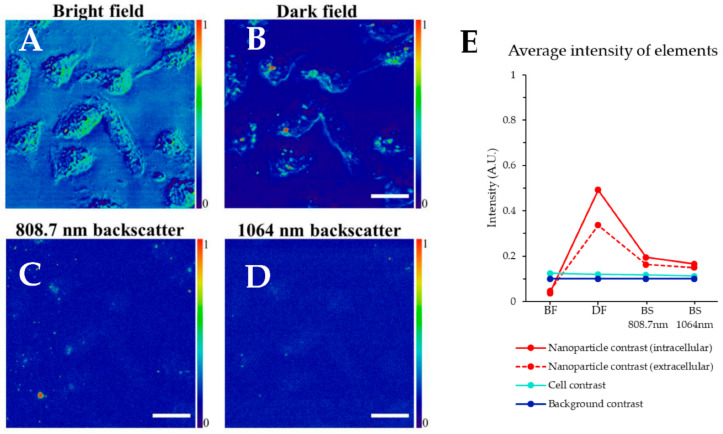
Single beam laser microscopy images of magnetic nanoparticles uptaken in GR-KLM-1 cells, (**A**) transmission bright field (BF) with 808.7 nm illumination, (**B**) transmission dark field (DF) with 808.7 nm illumination and (**C**) epi-detected backscatter (BS) with 808.7 nm, and 1064 nm illumination (**D**). All scale bars are 20 μm. (**E**) shows the average intensity of the various image elements, see legend, data is offset by and scaled to the background intensity to aid comparison, with error bars indicating one standard deviation for 10 measurements. Solid and dotted trendlines have been added to aid comparison.

Figure 2 shows a series of microscopy images of the 20.5 ± 1.3 nm Fe_3_O_4_ + ≈ 4 nm thick Au shell magnetic-plasmonic nanostructures uptaken in GR (gemcitabine resistant) human pancreatic cancer cells. Beginning with the laser bright field image (Figure 2A), we can see the cells (likely owing to focus conditions and illumination inhomogeneity) but cannot localize nanoparticles. Meanwhile, in the laser dark field (Figure 2B), nanostructures can be seen as higher intensity areas with respect to the background signal (in red), which is a result of significant light scattering owing to the plasmonic property of the nanostructure. Herein, we define an element’s contrast with respect to the background signal, unless otherwise stated. The edges of the cells also generate contrast due to light scattering, but inner areas show minimal contrast. In previous work, we show that the magnetic-plasmonic nanoparticles have a scattering peak wavelength of 625 ± 11 nm and may be imaged by laser dark field microscopy [21]. As the laser is tuned to 808.7 nm for these bright and dark field images, we do not expect peak scattering. However, using 808.7 nm may be preferable to ca. 625 nm for bioimaging due to minimized tissue absorption and scattering in the near-infrared biological imaging window [26]. The epi-detected backscatter images at 808.7 and 1064 nm are also shown in Figure 2C,D (CARS images use both illuminations simultaneously). Some nanoparticles can be located using epi-detected backscatter imaging, more so at 808.7 nm likely due to its proximity to the scattering peak of the nanostructures. However, cells cannot be visualized using backscatter imaging, see Figure 2C,D. Figure 2E shows the average intensity of the various elements including intracellular nanoparticles, extracellular nanoparticles, cells, and background intensity using different techniques—bright field (BF), dark field (DF) and backscatter (BS) at 808.7 and 1064 nm. For bright field, the cells have slightly higher average intensity compared to the background, while the nanoparticles have a lower intensity than the background. In dark field, the extracellular nanoparticle intensity is high, however, uptaken nanoparticles have a higher intensity. Cells and background have comparable intensity; however, the cell borders do scatter some light. For backscatter imaging at 808.7 and 1064 nm, the nanoparticles generate reasonably high contrast while cells do not. Hence, none of these techniques are ideal for combined nanoparticle-cell imaging.

**Figure 3:**
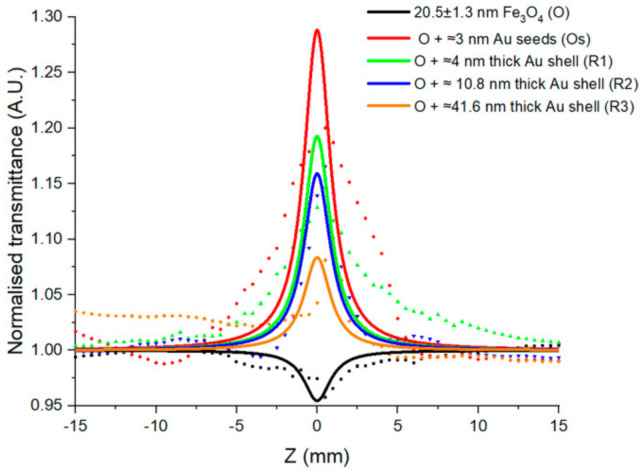
Open aperture transmission z-scan obtained for Fe_3_O_4_ nanoparticles and Fe_3_O_4_ nanoparticles with various thicknesses of gold. The solid line is the fit of the normalized transmittance expression of the experimental data points.

Meanwhile, CARS microscopy may be contrasted by the materials’ intrinsic molecular vibration. In biological samples like tissues and cells, we can consider three key molecular vibrations, at 2926 cm^−1^, 2850 cm^−1^ and 2967 cm^−1^ to probe protein, lipid, and DNA, see Table 1 [27]. The intensity of CARS signal is proportional to the square of the magnitude of the complex cubic nonlinear-optical susceptibility of the medium, χ^(3)^, which may be derived from z-scan measurements showing saturable and reverse saturable absorption [28]. Typically, the nonlinear susceptibilities associated with biological materials are small, but the frequency difference between the pump and Stokes beam can be tuned to probe Raman active bonds. Figure 3 shows the open aperture z-scan data for magnetic nanoparticles with different thicknesses of gold. Fe_3_O_4_ nanoparticles (seen in black in Figure 3) show a reverse saturable absorption (negative peak value), as observed previously in Fe_3_O_4_ nanoparticles [29].

We see an increased transmission at focus (z = 0) (positive peak value) for gold-coated magnetite nanoparticles, indicative of saturated absorption where the ground state absorption is higher than the excited state [22]. This saturated absorption in plasmonic systems is often referred to as plasmon bleaching, where the collective oscillation of conduction band electrons saturates. Saturated absorption has been observed using the open aperture z-scan technique of gold nanoparticles [30], Si-gold nanoshells [31], and even γ-Fe_2_O_3_-Au nanoparticles [32]. In this work, the peak decreases with increased thickness in gold, see Figure 3. Similar trends have been observed for gold shell thickness on silica nanoparticles in terms of the two-photon absorption cross-section, where the thinnest gold shells proved most effective [33]. Fitting this data (per [22]) allows the magnitude of the nonlinear absorption coefficient, β, and the imaginary part of the third-order susceptibility, Im χ^(3)^ to be extracted. This reveals a reduction in the magnitude β and Im χ^(3)^ with increased gold thickness, as seen in Table 2.

**Figure 4:**
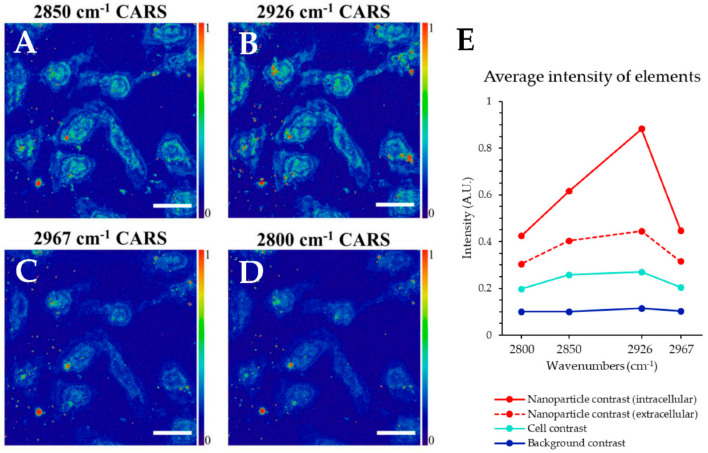
Series of epi-CARS images of magnetic-plasmonic nanoparticles uptaken in GR-KLM−1 cells at 2850 cm^−1^, 2926 cm^−1^, 2967 cm^−1^, and 2800 cm^−1^**.** All scale bars are 20 μm. (**E**) shows the average intensity of the various image elements for the different CARS wavenumbers with error bars indicating one standard deviation in a population of 10 measurements. Solid and dotted trendlines have been added to aid comparison.

Figure 4 shows a number of epi-detected CARS images of the 20.5 ± 1.3 nm Fe_3_O_4_ + ≈4 nm thick Au shell (R1) magnetic-plasmonic nanostructures uptaken in GR (gemcitabine resistant) human pancreatic cancer cells. Figure 4 includes an epi-CARS image probing at 2850 cm^−1^ associated with lipids; 2926 cm^−1^ vibration indicative of protein; 2967 cm^−1^ vibration, which is associated with DNA; and 2800 cm^−1^ was taken as a non-resonant background. We can see contrast associated with the cell in cyan, but the nanoparticles are strongly contrasting in red. These high intensity regions associated with nanoparticles are likely aggregations of nanoparticles. For an example of CARS imaging without nanoparticles, see Figure S1. CARS and DF microscopy are diffraction limited, and thus cannot resolve single nanostructures; if this is of interest, electron microscopy or super resolution optical techniques should be employed.

Figure 4E shows the average intensity of the various elements including intracellular nanoparticles, extracellular nanoparticles, cells, and background at the four probed CARS wavelengths—2800, 2850, 2926, and 2967 cm^−1^. The protein and lipid-associated CARS wavelengths are the best choices for cellular contrasting, see cyan points. Interestingly, the average intensity of intracellular nanoparticles is higher than that of nanoparticles outside of cells. Hence, it is likely that plasmon-associated surface-enhancement of the CARS signal is occurring to enhance the nanoparticle contrast. Moreover, the epi-detected scatter image and transmission dark field (Figure 2) imaging using only 808.7 nm excitation do not show as much nanoparticle-associated contrast as epi-CARS imaging. In previous work, we show that the magnetic-plasmonic nanoparticles have a scattering peak wavelength of 625 ± 11 nm, which is spectrally near the CARS wavelength in this work (652.2−666.7 nm, see Table 1).

**Figure 5:**
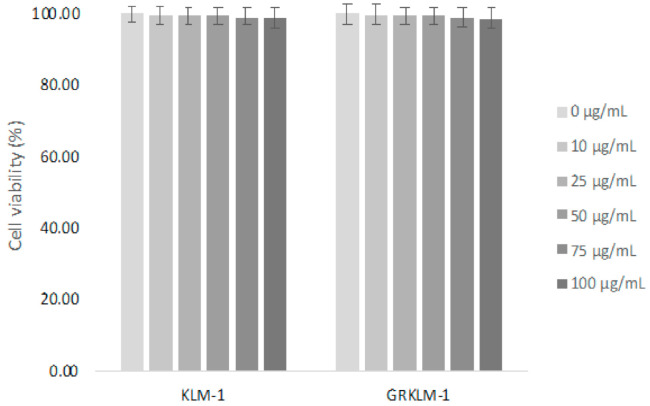
Biocompatibility studies of magnetic-plasmonic nanoparticles of various concentrations in KLM-1 and GR-KLM-1 cell lines.

Figure 5 shows cell viability studies, demonstrating good tolerability of the magnetic-plasmonic nanoparticles in two human cell lines KLM-1 and GR-KLM-1. No significant impact on cell viability for nanoparticle concentrations of 10–100 μg/mL was observed, as calculated using One-Way ANOVA with post-hoc analysis via the Dunnett method using Minitab (*p* < 0.05). Good biocompatibility is important to ensure non-targeted tissue (outside the targeted area) is not damaged with cells alone [34,35] and that cell death is only initiated by use of external stimuli [36]. Espinosa et al. [37] discussed the effect of internalization of nanoparticles (incubation time) in magnetic hyperthermia and photothermal applications, finding that cellular internalization led to a marked decrease in magnetic hyperthermia but photothermal activity was seen to increase upon uptake.

## 4. Conclusions

Magnetic-plasmonic, Fe_3_O_4_-Au, core-shell nanoparticles were shown to be highly biocompatible in both human pancreatic cancer cell line (KLM-1) and the gemcitabine resistant cell line (GR-KLM-1). This biocompatibility is vital as Fe_3_O_4_-Au nanoparticles are typically selected to be inactive until an external stimulus like light or an alternating magnetic field is applied [36]. Broadband dark field microscopy is a common tool used for visualization of plasmonic nanoparticles, but the lack of contrast originating from biological materials and the visible light wavelengths used means that it is not an effective technique.

Herein, we discuss the use of epi-CARS of magnetic-plasmonic nanoparticles in cells. First, we explore the nonlinear optical properties of various gold shell thicknesses on a magnetic core nanoparticle using z-scan techniques, finding that bare iron oxide nanoparticles demonstrate reverse saturable absorption while gold-coated nanostructures exhibited saturable absorption, with the calculated nonlinear absorption coefficient (β) and imaginary part of the third-order susceptibility (Im χ^(3)^) decreasing with increased gold shell thickness. Thin gold shells and gold seeded iron oxide nanoparticles showed the highest magnitude of the imaginary part of the third-order susceptibility, suggesting their suitability as contrast agents in nonlinear optical microscopy.

Next, we find that the epi-CARS imaging technique is superior to standard dark field imaging for nanoparticle visualization, owing to improved nanoparticle contrast, while simultaneously generating cellular contrast. We show improved nanoparticle contrast compared to laser dark field and epi-detected backscatter images. Moreover, near-infrared light may be utilized for imaging, which is more suitable for bioimaging while also avoiding the photothermal effects at the absorption peak of the nanostructure, allowing imaging while minimizing unintended nanoparticle heating.

## Figures and Tables

**Table 1:** The probed vibrations in CARS and the wavelengths involved when using a Stokes beam at 1064 nm.

Target	Stretch	Wavenumbers	Pump Wavelength	CARS Wavelength
DNA	CH	2967 cm^−1^	808.7 nm	652.2 nm
Protein	CH_3_	2926 cm^−1^	811.4 nm	655.7 nm
Lipid	CH_2_	2850 cm^−1^	816.4 nm	662.3 nm
Background	Off resonance	2800 cm^−1^	819.8 nm	666.7 nm

**Table 2:** Various magnetic-plasmonic nanostructures and their associated nonlinear absorption parameters*—*β is the nonlinear absorption coefficient and Im χ^(3)^ is the imaginary part of the third-order susceptibility.

Nanoparticle	β (10^−10^ m/W)	Im χ^(3)^ (10^−12^ e.s.u.)
20.5±1.3 nm Fe_3_O_4_ (O)	1.2	1.1
O + ≈3 nm Au seeds (Os)	−7.4	−5.7
O + ≈4 nm thick Au shell (R1)	−4.9	−3.9
O + ≈ 10.8 nm thick Au shell (R2)	−4.1	−3.2
O + ≈41.6 nm thick Au shell (R3)	−2.1	−1.7

## Data Availability

Not applicable.

## Supplementary Materials

The following are available online at https://www.mdpi.com/2079-4991/11/3/685/s1, Figure S1: CARS at 2926 cm^−1^ of GR-KLM-1 cells without nanoparticle exposure, with a notable absence of high intensity spots associated with nanoparticles.
