# Supplementary material for: Dark Field and Coherent Anti-Stokes Raman (DF-CARS) Imaging of Cell Uptake of Core-Shell, Magnetic-Plasmonic Nanoparticles
Source: Nanomaterials (Basel). 2021 Mar 9;11(3):685. doi: 10.3390/nano11030685 (PMC7998699; doi:10.3390/nano11030685)
Supplement: Supplementary file 1 [file nanomaterials-11-00685-s001.pdf]

nanomaterials

MDPI

Supplementary Materials

Dark field and Coherent Anti-Stokes Raman (DF-CARS) Imaging of Cell Uptake of Core-Shell, Magnetic-Plasmonic Nanoparticles

Grace Brennan [1], Sally Ryan [2], Tewfik Soulimane [2], Syed A. M. Tofail [1] and Christophe Silien [1,*]

[1] Department of Physics and Bernal Institute, University of Limerick, V94 T9PX Limerick, Ireland; Grace.Brennan@ul.ie (G.B.); Tofail.Syed@ul.ie (S.A.M.T.)
[2] Department of Chemical Sciences and Bernal Institute, University of Limerick, V94 T9PX Limerick, Ireland; Sally.Ryan@ul.ie (S.R.); Tewfik.Soulimane@ul.ie (T.S.)
* Correspondence: Christophe.Silien@ul.ie

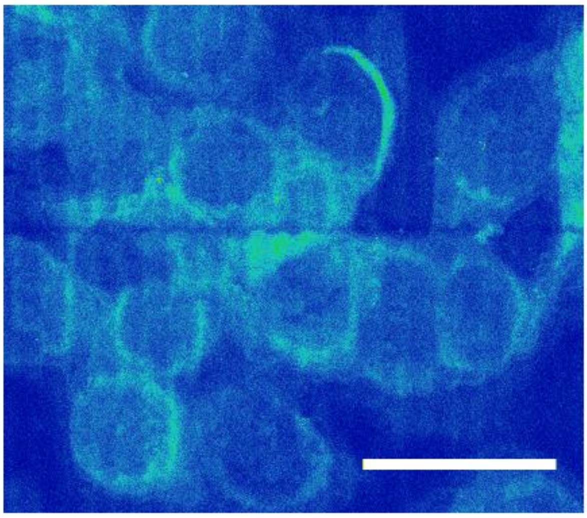

Figure 1. CARS at 2926 cm^{-1} of GR-KLM-1 cells without nanoparticle exposure, with a notable absence of high intensity spots associated with nanoparticles. Scale bar is 20 µm.
